# Recent advances in microscopic techniques for visualizing leukocytes *in vivo*

**DOI:** 10.12688/f1000research.8127.1

**Published:** 2016-05-19

**Authors:** Rohit Jain, Shweta Tikoo, Wolfgang Weninger

**Affiliations:** 1 Immune Imaging Program, The Centenary Institute, University of Sydney, Newtown, NSW 2042, Australia; 2 Discipline of Dermatology, Sydney Medical School, University of Sydney, NSW 2006, Australia; 3 Department of Dermatology, Royal Prince Alfred Hospital, Camperdown, NSW 2050, Australia

**Keywords:** leukocytes, visualizing, in vivo

## Abstract

Leukocytes are inherently motile and interactive cells. Recent advances in intravital microscopy approaches have enabled a new vista of their behavior within intact tissues in real time. This brief review summarizes the developments enabling the tracking of immune responses *in vivo*.

## Introduction

The elicitation of an immune response against invading pathogens or tumor cells relies on a complex and highly orchestrated interplay among leukocytes, blood vessels, and stromal cell populations. During this process, immune cells need to identify and interpret structural and molecular guidance cues such as extracellular matrix fibers and chemokines to navigate through diverse tissue microenvironments towards foci of tissue injury, where they finally mediate their effector functions. Microscopic investigations into this highly orchestrated behavior of immune cells have provided insight into the workings of innate and adaptive immunity in live mice at a level of resolution previously unattained.

To visualize the behavior of immune cells *in vivo,* initial studies relied on bright field and epifluorescence video microscopy. Although limited in resolution and restricted by tissue clarity, these studies were instrumental for dissecting the molecular mechanisms regulating leukocyte homing during the process of extravasation ^1– 4^. However, the advent of advanced confocal ^5^ and two-photon ^6, 7^ imaging modalities revolutionized our understanding of lymphocyte behavior within the interstitial space of intact organs, initially within re-aggregated thymic organ cultures ^6^ and explanted intact lymph nodes ^5, 7^. These studies illuminated the complex *in situ* behavior of T cells within lymph nodes in the absence or presence of antigen ^5, 7^ and during their selection within the thymus ^6^. These advancements in imaging technologies further catalyzed the emerging field of intravital imaging of the immune system. Indeed, shortly after the explant models, imaging of intact organs *in situ* in living mice was reported ^8, 9^. Since then, a large number of tissues/organ systems have been adapted for *in vivo* imaging, revealing insights into the functioning of the immune system in the steady state, during initial pathogen encounter, during the priming phase of adaptive immune responses, and within effector organs during infection, inflammation, and within tumors. The impact of intravital imaging on the field of leukocyte biology and immunology has recently been reviewed in detail elsewhere ^10– 13^.

In summary, the development of new imaging tools and molecular probes, coupled with a wealth of transgenic fluorescent reporter mice, has ushered in a new era for understanding the cellular and molecular regulators of leukocyte function *in situ*. In this review, we highlight some of the technical advances in this field of research, with a focus on multi-photon imaging.

## Advances in two-photon imaging methodologies

Without doubt, the introduction of two-photon microscopy into biological imaging has enormously furthered our understanding of physiological and pathological processes at the level of intact organs. Unlike single-photon excitation used in confocal microscopy, two-photon excitation is spatially restricted to the focal plane ^10, 12, 14, 15^. This results in reduced levels of phototoxicity and photobleaching, enabling deep tissue imaging for prolonged periods of time ^10, 12^. Nevertheless, several technical challenges still persist. For example, physiological tissue movement due to pulsation of blood vessels, contraction of muscles, respiration, or heartbeat can be detrimental to the overall quality of the data. In addition, surgical and anesthetic requirements may alter the behavior of cells due to the induction of hypoxia, changes in tissue pH, or the generation of pro-inflammatory mediators. It is therefore imperative to develop standardized, reproducible procedures for the exposure of organs during the imaging session.

For many peripheral tissues like skin ^16^ and draining lymph nodes ^8, 9, 17^, and internal organs such as the liver ^18– 21^ and spleen ^22– 24^, tissue stabilization during imaging has been carried out using relatively simple methodologies and devices. Similarly, intravital imaging of bone marrow cavity within the skull calvaria has been adapted for two-photon microscopy ^25^ and has played a significant role in highlighting immune cell behavior within this environment ^25– 28^. Akin to this, various methodologies have been developed for longitudinal imaging of tumors ^15^. Cumulatively, these new imaging methodologies have provided unprecedented understanding of leukocyte behavior *in vivo* in a variety of tissues ^11– 13, 29– 31^.

Nonetheless, stable imaging of certain tissues, such as the brain, heart, and lung, still presents a significant challenge. In the case of the brain, the two most frequently used methodologies involve either bone thinning ^32, 33^ or removal of the cranial bone ^34– 36^ for imaging of the underlying meninges and brain parenchyma. Similarly, longitudinal imaging of the brain has been made possible by implantation of an optical window ^34, 37, 38^. These methodologies allow studying the behavior of various immune cells like microglia ^36, 39, 40^, monocytes ^41, 42^, and T cells ^13, 43– 46^ within the intact brain and spine under homeostasis, infection, or inflammation. Newer techniques, such as implantation of glass prisms ^47^ or micro-optical probes ^48^, have been developed for imaging of deeper structures within the brain ^47, 48^, but they are yet to be harnessed for understanding leukocyte behavior.

*In vivo* imaging of lungs presents a significant technical challenge due to tissue movement and its localization within the pleural cavity. Nonetheless, several strategies have been developed that involve the closing of ventilation to the imaged lung ^49^ or synchronizing image acquisition with respiration ^50^. Recently, several new methodologies ^51– 53^ have described the creation of a small thoracic window, wherein the tissue is stabilized via application of a mild negative pressure ^51, 52^ or surgical glue ^53^. These approaches maintain ventilation and perfusion within the tissue and permit imaging throughout the respiratory cycle ^51, 52^. These recent advances have highlighted the dynamic nature of T cells ^51, 54^, neutrophils ^28, 51, 53^, monocytes ^53^, macrophages ^55^, and dendritic cells ^54, 55^ within the pulmonary tissue in the steady state and during inflammatory conditions. For instance, *in vivo* imaging of the lungs during influenza virus infection has identified a unique cooperative behavior between neutrophils and CD8 ^+^ effector T cells. Thus, within influenza-infected lungs, migrating neutrophils leave behind a trail of the chemokine CXCL12, which is used by effector CD8 ^+^ T cells as a guidance cue within the interstitium ^56^. This study enforces the role of *in vivo* imaging in understanding the dynamic spatiotemporal cooperation between immune cells required for generating an efficient immune response.

By using endoscopic time-lapse imaging ^57^ or optical windows ^58– 60^, researchers have recently addressed the challenges associated with *in vivo* imaging of leukocytes within the beating heart ^61^. Endoscopic imaging of the heart under physiological conditions revealed the patrolling behavior of monocytes within coronary vessels ^57^. Post-infarction, rapid recruitment of both monocytes and neutrophils was observed ^57, 58^. Surprisingly, unlike most tissues, large coronary veins but not post-capillary venules ^13^ were the predominant sites for neutrophil extravasation post-ischemia/reperfusion injury ^58^. Most neutrophils migrated via “hotspots” within the inflamed coronary veins ^58^. Although this technique is still in its early days, *in vivo* imaging of the heart nonetheless holds immense potential in understanding leukocyte/lymphocyte behavior post-cardiac transplant and during organ rejection.

In addition to the intravital imaging of the beating heart, several methodologies have been developed to image blood vessels *in vivo* ^62– 65^. Studies have highlighted, for example, the dynamic migratory behavior of neutrophils ^63, 64^ and T lymphocytes ^63^ within atherosclerotic lesions. Recently, the use of cardiac triggered acquisition coupled with image registration and post-processing image correction has provided further advancement of stable imaging of blood vessels ^65^. This methodology enables improved temporal resolution, which has been used to define the dynamic scanning behavior of dendritic processes of leukocytes within the atherosclerotic plaques of pulsatile blood vessels ^65^.

The use of two-photon excitation can also be exploited for inducing localized injury without physical manipulation of the surrounding tissue. This provides opportunities for studying the behavior of tissue-resident and recruited leukocytes during sterile inflammation *in situ*. This methodology has been used, for example, to highlight the dynamic behavior of microglia within the brain post injury ^36, 39^ and to delineate the cellular and molecular players required for interstitial migration of neutrophils ^66, 67^, monocytes ^67^, and dendritic cells ^68^ within the skin ^13, 69, 70^. Thus, laser-induced injury has been crucial for understanding the cellular and molecular interplay required for interstitial migration of neutrophils and the damage response within the inflamed dermis ^66, 67^. Following injury, scarce initial neutrophils rapidly migrate towards the site of injury following chemokine gradients. This has been dubbed the “neutrophil scouting phase”, which is followed by an “amplification phase” wherein a large number of neutrophils display directed migration towards the injury site. This second phase is dependent on cyclic-adenosine diphosphate ribose (c-ADPR) and leukotriene B4 (LTB4) signaling ^66, 67^. The large influx of neutrophils at the injury site leads to the formation of a stable neutrophil cluster also referred to as the “stabilization phase”. The stability of the cluster is governed by signaling via G-protein-coupled receptors such as C-X-C chemokine receptor 2 (CXCR-2), *N*-formyl peptide receptor 2 (FPR-2), and LTB4 receptor-1 (BLT-1). Surprisingly, although integrins were dispensable for neutrophil migration within the inflamed dermis, they were required for migrating within the injury foci devoid of extracellular matrix. Together, these studies uncover the crucial role of intravital imaging in understanding the spatiotemporal dynamics of the innate immune response *in vivo*.

The use of *in vivo* imaging has also been instrumental for understanding immune responses against invading pathogens ^13, 71, 72^ and has highlighted the dynamic behavior of innate and adaptive immune cells during various infections including *Leishmania major* ^73– 77^, *Leishmania donovani* ^78– 80^, *Toxoplasma gondii* ^43, 81– 83^, *Borrelia burgdorferi* ^84, 85^, *Staphylococcus aureus* ^86– 88^, *Plasmodium spp.* ^42, 46, 89– 93^, and *Mycobacterium-*induced granulomas ^19, 21^. These studies have identified various strategies used by pathogens to evade immune responses; for instance, post-intradermal inoculation, *Plasmodium berghei* sporozoites rapidly migrate into the blood vessels and lymphatics ^89^. In contrast, experiments performed with another vector-borne parasite, *Leishmania major*, revealed that these parasites do not actively migrate but rather induce neutrophil recruitment to aid parasite survival ^73, 74, 94– 97^. Similarly, *in vivo* imaging of mycobacterial granulomas within the liver have highlighted the presence of numerous antigen-presenting cells (APCs) and CD4 ^+^ T cells within the granulomatous tissue ^19, 21^. A paucity of mycobacterial antigens limited the number of stable interactions between APCs and antigen-specific CD4 ^+^ T cells and thereby suppressed the release of immunomodulatory/protective cytokines ^21^. Cumulatively, *in vivo* imaging of host-pathogen interactions have identified novel cellular and molecular mechanisms involved in regulating pathogen uptake and transport, generation of adaptive immune responses, identification of new migratory strategies used for scanning infected tissues, and elicitation of effector immune responses.

Similarly, two-photon imaging of tumors has provided fascinating insights into leukocyte behavior within the tumor milieu: for example, effector CD8 ^+^ T cell-mediated scanning of tumor tissue ^98– 100^, induction of tumor cell apoptosis ^101^, generation of T cell tolerance ^102^, co-migration of macrophages and tumor cells ^103^, and the role of perivascular macrophages in tumor cell dissemination ^104^. Using experimental models that recapitulate the pathophysiology of multiple sclerosis, investigators have revealed fibrinogen-mediated perivascular clustering of microglia ^40^, formation of immunological synapse between APCs and effector CD4 ^+^ T cells ^44^, and neuronal cell death ^105^ with the use of *in vivo* imaging.

The latest development in the use of optical phase-locked ultrasound lens provides a new methodology for high-speed volumetric imaging *in vivo* ^106^. This allows for the imaging of Ca ^2+^ signaling as well as alterations in cell morphology at high speeds ^106^. This methodology has been used to highlight intravascular and extravascular neutrophil dynamics at a temporal resolution previously unattainable ^106^.

Altogether, the advancements in imaging instrumentation and development of new and improved physiological models have been crucial in developing a deeper and dynamic understanding of innate and adaptive immunity.

## Transgenic fluorescent reporter mouse models for *in vivo* imaging

Visualization of cells and structures within tissues via laser excitation relies, on the one hand, on their inherent (auto)-fluorescent features; for example, second and third harmonic generation facilitate highlighting large extracellular molecules such as collagen and elastin fibers without the need for counterstaining ^107– 110^. In addition, certain auto-fluorescent metabolites such as NADPH enable the interrogation of metabolic states of cells ^111, 112^. On the other hand, most leukocyte subsets require external or genetic tagging for their detection. Initial imaging studies made use of *ex vivo* labeled, adoptively transferred immune cells. Although manipulation of cells may alter their behavior, these studies were crucial in understanding migratory and interactive cellular dynamics *in vivo*. Nevertheless, advances in genetic engineering technology coupled with the availability of a wide array of genetically encoded fluorophores have significantly expanded the scope of *in vivo* imaging. Introduction of transgenic fluorescent proteins at specific genetic loci or under the control of tissue- or cell-specific promoters has been crucial for following cells of interest under physiologic conditions. Numerous fluorescent reporter mouse strains are now available for *in vivo* imaging, and the choice of a particular reporter relies on the experimental design and the scientific question being pursued ^113, 114^. Although the detailed description of these strains is beyond the scope of this review, a few of the novel and versatile reporter mouse strains are detailed below.

The dynamic nature of immune cells presents a significant challenge while tracking the individual cell longitudinally. This challenge can be overcome by using photoactivatable fluorescent reporters, for example photoactivatable GFP ^115^. Using such an approach, Victora and colleagues deciphered B cell ^116^ and T follicular helper cell ^117^ dynamics within the germinal center of murine lymph nodes, providing valuable information regarding the regulation of humoral immunity. This strategy is useful for tracking the migration and distribution of immune cells *in vivo.* However, the degradation of photoactivated GFP within the marked cells limits the timespan available for tracking, usually in the order of days (t _½_ - 30 hours for naive B cells ^116^). More recently, photo-switchable transgenic reporters like KikGR ^118, 119^ and Kaede ^120^ mice have become available. Briefly, in Kaede mice, ubiquitously expressed photoconvertible GFP ^121^ undergoes peptide cleavage and alteration within the chromophore upon exposure to ultraviolet light, resulting in a shift from green to red fluorescence ^120^. This conversion is irreversible and thereby marks the treated cell until the photoconverted Kaede is degraded and replaced by new Kaede protein ^120^. Kaede mice have been used to decipher the migratory kinetics and behavior of T follicular helper cells under homeostasis or during memory responses ^122^. In brief, the above-mentioned strategies coupled with *in vivo* imaging have revealed the dynamic behavior of T follicular helper cells during primary and secondary immune responses. During the primary response, T follicular helper cells are restricted within the germinal center, where they provide cognate help to developing B cells. However, during secondary immune responses (akin to a recurrent infection), the memory T follicular helper cells show unrestricted migration in and out of the germinal centers, a strategy that might be useful in eliciting rapid humoral responses ^122^.

Although both KikGR and Kaede mice have been used for tracking the dynamics of various immune cell subsets like neutrophils ^123^, T cells ^124, 125^, innate lymphoid cells ^126^, and dendritic cells ^127^ at a population level, great opportunities still persist in understanding the behavior of these immune cells at single-cell resolution.

Another strategy to irreversibly mark individual cells *in vivo* relies on the Cre/lox recombination system. Using this strategy, Livet and colleagues generated the Brainbow mouse strain where Cre-mediated recombination stochastically permutates multiple copies of the construct containing several different fluorescent proteins ^128, 129^. This recombination strategy results in a mosaic of fluorescent colors leading to the detection of nearly 90 separate colors that can be used to mark individual cells. This strategy was initially used to identify individual neurons within the CNS and for tracing neuronal circuitry ^128^. The generation of the Brainbow mouse paved the way for the development of several new approaches like LeGO ^130^, Confetti ^131^, and Ubow ^132^ to mark cells for tracing the fate of individual cells. Ubow mice have been used for fate mapping of Langerhans cells ^132^ and follicular dendritic cells ^133^ within the skin and lymph node, respectively. Recently, a combination of fluorescent reporters and the Cre/lox system has been used exquisitely to highlight *in vivo* transfer of metastatic information between tumor cells ^134^. Use of the Cre/lox system provides a significant advantage over other strategies such as photoactivation or photoconversion by genetically/permanently labeling the cells, thereby enabling tracking throughout the lifetime of the cell. However, in most scenarios, the genetic labeling is targeted to a subset of cells and not to spatiotemporally selected individual cells.

Taken together, the recent advances in recombinant DNA technology like TALEN and the CRISPR/Cas9 genome editing system along with the availability of a multitude of reporter and knockout mouse strains promise a more colorful and dynamic future for the *in vivo* imaging of leukocytes.

## Other methodologies

Although the use of transgenic fluorescent reporter strains for *in vivo* imaging has been gaining momentum, the transduction of immune cells using various fluorescent reporters still provides a valuable tool for understanding leukocyte function. These strategies have been used to delineate activation ^45, 102, 135^, calcium signaling ^136^, and apoptosis ^137^ within T cells *in vivo*. Similarly, several fluorescent probes are available to highlight cellular and molecular events crucial for leukocyte function *in vivo*. These fluorescent probes have been utilized to decipher various cellular processes: for example, NETosis ^88, 138^, the generation of reactive oxygen species ^139^, and cell death ^67, 140^. The use of fluorescent probes to mark DNA coupled with *in vivo* imaging has been crucial in deciphering the physiological role of DNA-NETs (DNA-neutrophil extracellular traps). Intravital imaging in various infectious model systems has uncovered the important role of DNA-NETs in ensnaring and killing pathogens ^88, 141– 143^. Although DNA-NETs are an essential arsenal of the innate immune response, release of DNA-NETs *in vivo* can trap circulating tumor cells and enhance metastasis ^144^.

Likewise, novel approaches have been developed to couple immune cell behavior with receptor-based signaling *in vivo* via administration of labeled antibodies. In this methodology, labeled Fab fragments are administered *in vivo* and the distribution of cell surface receptors and the behavior of labeled cells are recorded, segmented, and analyzed as flow cytometry plots ^145^. This methodology is of immense importance where the migratory dynamics of immune cells need to be analyzed in context with the receptor signals delivered to individual immune cells *in situ*.

## Concluding remarks

In the past decade, *in vivo* two-photon imaging has provided researchers with unparalleled views of immune cell behavior under homeostasis or pathology. This has resulted in the identification of new molecular and cellular regulators of the immune system.

In our laboratory, *in vivo* imaging has been instrumental in highlighting the dynamic behavior of neutrophils ^66^, monocytes ^42^, T cells ^146, 147^, dendritic cells ^73^, type-2 innate lymphoid cells ^148^, and perivascular macrophages ^86^ under homeostasis or during various infections or inflammatory conditions.

Nonetheless, the use of *in vivo* imaging to understand cell-to-cell communication and the generation of complex interaction networks still requires extensive investigation. Intravital multiphoton imaging in most tissues is restricted to approximately 500 μm from the surface, which limits the visualization of processes within deeper tissues *in vivo*, therefore leaving certain aspects of the immune response in the dark. Various other strategies such as optical frequency domain imaging (OFDI) ^149^ and speckle-variance optical coherence tomography (svOCT) ^150^ have been developed for overcoming this limitation; however, these methodologies currently lack the necessary resolution for imaging immune cell behavior and are mostly useful for imaging tissue architecture and the vasculature ^151, 152^.

We believe that the increased availability of fluorescent reporters for *in vivo* marking of cellular and molecular signaling, technical improvements in instrumentation (for example, adaptive optics), and better analytical capability will be crucial for understanding the complex behavior of leukocytes *in vivo*.
